# Continuous Space Estimation: Increasing WiFi-Based Indoor Localization Resolution without Increasing the Site-Survey Effort †

**DOI:** 10.3390/s17010147

**Published:** 2017-01-13

**Authors:** Noelia Hernández, Manuel Ocaña, Jose M. Alonso, Euntai Kim

**Affiliations:** 1 Intelligent Systems Laboratory, Department of Systems Engineering and Automation, Universidad Carlos III de Madrid, 28911 Leganés, Madrid, Spain; 2 Robesafe Research Group, Department of Electronics, Universidad de Alcalá, 28871 Alcalá de Henares, Madrid, Spain; mocana@depeca.uah.es; 3 Centro Singular de Investigacion en Tecnoloxias da Informacion (CiTIUS), Universidade de Santiago de Compostela, Campus Vida, E-15782, Santiago de Compostela, Galicia, Spain; josemaria.alonso.moral@usc.es; 4 Computational Intelligence Lab, School of Electrical and Electronic Engineering, Yonsei University, 03722 Seoul, Korea; etkim@yonsei.ac.kr

**Keywords:** WiFi indoor localization, fingerprinting, continuous space estimation, machine learning, location-based services

## Abstract

Although much research has taken place in WiFi indoor localization systems, their accuracy can still be improved. When designing this kind of system, fingerprint-based methods are a common choice. The problem with fingerprint-based methods comes with the need of site surveying the environment, which is effort consuming. In this work, we propose an approach, based on support vector regression, to estimate the received signal strength at non-site-surveyed positions of the environment. Experiments, performed in a real environment, show that the proposed method could be used to improve the resolution of fingerprint-based indoor WiFi localization systems without increasing the site survey effort.

## 1. Introduction

Location-Based Services (LBSs) can be defined as the services that integrate a mobile device’s location or position with other information to provide added value to the user. As part of its annual Mobile Life study of 2012 [1], TNS found that 19% of the world’s six billion mobile users were already using LBSs, with 62% of the non-LBS users aspiring to do so in the future. They have also found that navigation was the most popular motivation behind LBSs (46%). However, besides the classic navigation, the use of LBSs allows for applications in very different areas: social networking [2], healthcare [3,4], guidance in museums or public buildings [5], security [6], intelligent transportation systems [7], logistics [8], etc.

Traditionally, global localization has been carried out through the Global Positioning System (GPS) [9], which provides accurate localization when working outdoors. This way, GPS has become the main technology for positioning in outdoor environments. Encouraged by the accuracy of the GPS outdoors and due to the fact that almost every new smartphone and tablet has built-in GPS receivers, most of the currently-existing LBSs are oriented to outdoor environments. However, in indoor environments, LBSs are of equal interest in a wide range of personal and commercial applications.

All of these applications require accurate indoor localization for the strategic planning of the navigation or to provide guidance to the final target. Unfortunately, the use of GPS is affected by non-line-of-sight (satellite signals are attenuated and scattered by roofs, walls and other objects), making GPS localization in indoor environments not suitable. As a consequence, providing indoor localization requires the use of other technologies.

Different technologies are being used to provide indoor localization: infrared [10], ultrasound [11], laser [12], computer vision [13], radio frequency [14,15,16], the Global System for Mobile Communications (GSM) [17] or Li-Fi [18].

The decision about using one of these technologies is mainly determined by the accuracy required by the final application and the cost of the system deployment. The required accuracy for indoor LBSs is usually in the order of meters [19].

Some of the previously-named technologies, such as ultrasound or laser, may accomplish the accuracy requirements, but they require the use of additional hardware. Some others, such as ultra-wide band [20] or Radio Frequency Identification (RFID) [21,22], require the installation of artificial marks in the environment, increasing the cost of the system and forcing a specific deployment on every target environment.

If the goal is to provide a generic location service available on any device (smartphones, tablets, laptops, etc.) and in any environment, an already available technology in both the devices and the environments should be used. With this requirement in mind, a major group of indoor positioning systems utilizes radio frequency signals emitted by common wireless communication networks. Among all of the technologies, WiFi is arising as the most popular one. This is probably due to the advantages of using WiFi for indoor localization: WiFi Access Points (APs) are deployed in almost every building, and measuring the WiFi signal is free of charge even for private networks. This fact allows one to install a localization system based on WiFi without doing any modifications in the environment. Moreover, almost every device is already equipped with a WiFi interface, and no special requirements are needed to perform the localization. This way, almost every device can benefit from indoor LBSs using WiFi.

Among all indoor WiFi localization systems, fingerprint-based ones are some of the most popular ones. This kind of system uses a fingerprint database stored during a training stage to obtain the estimated position of the device by means of different classification algorithms [14,15,23]. The fingerprint database stores information, typically the Received Signal Strength (RSS), at certain locations of the environment, modeling the characteristics of the signal. The main disadvantage using this kind of system is the necessity of site survey for the fingerprint database construction, especially if the required resolution is high (which means more cells to site survey).

To reduce this problem, in this paper, we propose an approach to increase the resolution of fingerprint-based methods without the need for increasing the number of positions where fingerprints are collected and, as a consequence, the time required to build the database. This approach uses a Support Vector Regression (SVR) algorithm that estimates the RSS from the different APs at the coordinates of the environment that have not been site surveyed. Using these estimations, a fingerprint database composed of surfaces is created and used during the localization stage.

This paper is an extended version of the work published in the Proceedings of the International Conference on Indoor Positioning and Indoor Navigation [24]. We extend our previous work by improving the proposed method, including a new noise margin to reduce the localization error. In addition, deeper experimentation is provided, including a thorough analysis and validation of the method. We have also studied the map resolution and the number of topological positions required to train the system.

The remainder of the paper is organized as follows: First, Section 2 will provide a revision of the most significant research on WiFi indoor localization. Section 3 will describe the proposal. Then, the experimental results will be exposed in Section 4. Finally, a critical discussion about the results and the related future work will be provided in Section 5.

## 2. Related Work

Indoor localization has been one of the most active fields of research for the last decade. However, as was mentioned in the Introduction, WiFi indoor localization is still an open problem. At present, there are some available indoor localization systems based on WiFi. One of the most famous ones is the Google localization service, which combines GPS, WiFi and GSM with indoor Google Maps [25] to provide positioning in buildings, but its accuracy is not enough to provide an indoor guidance service yet. Other systems, such as the Ekahau positioning engine [4], rely on specially-designed hardware. These kinds of purpose-built systems can be expensive and hard to implement on a world-wide scale. As a consequence, a software-based algorithm should be used with the objective of providing a universal WiFi localization system.

Given the diversity of WiFi localization methods published in the literature, different classifications of them are possible [26]. Even though classification of some of the methods is not evident and there is a certain degree of overlapping between groups, they can be classified in terms of the algorithms that are used to solve the localization problem. Using this criterion, the studies can be separated into the following categories:

- Deterministic:
  - Propagation model based: Localization is carried out estimating the distance to nearby APs by means of a WiFi signal propagation model [27,28,29,30,31,32]. Propagation models describe how the signal is propagated in the environment, and they are used to translate the RSS into a distance. The APs’ location in the environment is normally known a priori. Using the distance to the APs and their positions in the environment, the typical choice is to use lateration algorithms to perform the localization.
  - Fingerprint based: These systems use a fingerprint database stored in a training stage to obtain the estimated position of the device by means of different classification algorithms [14,15,16,33]. The fingerprint database stores information, typically the RSS, at certain locations of the environment, modeling the characteristics of the signal using either discrete (fingerprints) or continuous (surfaces) representations.
- Probabilistic: These methods keep track of the position of the device maintaining a probability distribution over the positions or coordinates of the environment [34,35,36,37].

On the one hand, propagation model-based systems are the most precise ones if the propagation model is well adjusted for the environment. Unfortunately, this is hard to achieve indoors, being the main reason for the low acceptance of this kind of system for WiFi indoor localization. Theoretically, one propagation model could be used for localization in different environments; this is true when working outdoors, but indoors, it is usually necessary to do some site survey to adjust the model, loosing their main advantage.

On the other hand, fingerprint-based methods are valid only for the environment where they were trained, but with the necessary training stage, they can be used in environments of any characteristics.

The main disadvantage using this kind of system is the necessity of site surveying for the fingerprint database construction, especially if the required resolution is high (which means more cells to site survey). However, some systems are recently arising to reduce the site survey effort. Some of them focus on the automation of the site survey task [38,39,40,41], while other systems try to reduce the site survey effort by estimating the expected RSS at non-site-surveyed positions. Among the second group of methods, two approaches are starting to be used: interpolation using regression methods, such as Gaussian Process (GP) regression [42,43], or the creation of virtual fingerprints through the use of propagation models [44,45]. The use of propagation models to estimate the expected RSS at non-site-surveyed positions comes with some problems: it is necessary to adjust them for every environment in order to obtain good RSS estimations and to know the exact position of the APs, which sometimes, as is our case, is not possible to know.

To the best of our knowledge, our approach is the first to use support vector machines to interpolate the expected RSS at non-site-surveyed positions, reducing the effort of constructing a fingerprint-based WiFi localization system and providing a way of reducing the site survey effort without the need for adjusting propagation models or the need to know the location of the APs.

## 3. Continuous Space Estimator

**Figure 1:**
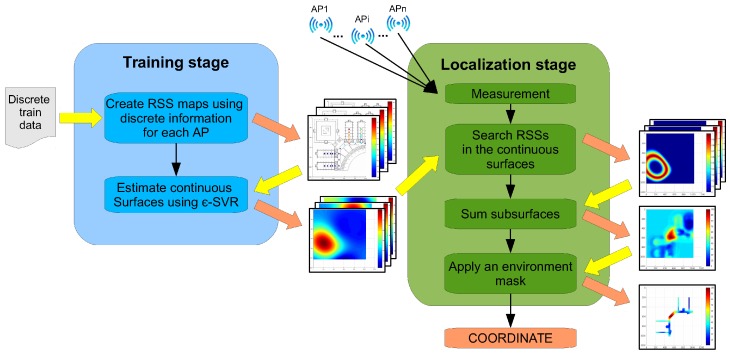
General architecture of the system.

This section presents a description of the Continuous Space Estimator (CSE) proposed to increase fingerprint-based methods’ resolution without increasing the site survey effort. The main objective is to infer the RSS from the existing APs in positions of the environment where no training data are available. To do so, the system will create RSS reference surfaces for each AP using an SVR algorithm [46] to estimate the missing data. Then, during the localization stage, the measured RSS from each AP will be searched in the corresponding surface. This way, the coordinate where the device is located will be estimated as the one found in the higher number of surfaces. A block diagram of the entire system is shown in Figure 1. This block diagram will be thoroughly explained in the next subsections.

### 3.1. Training Stage

**Figure 2:**
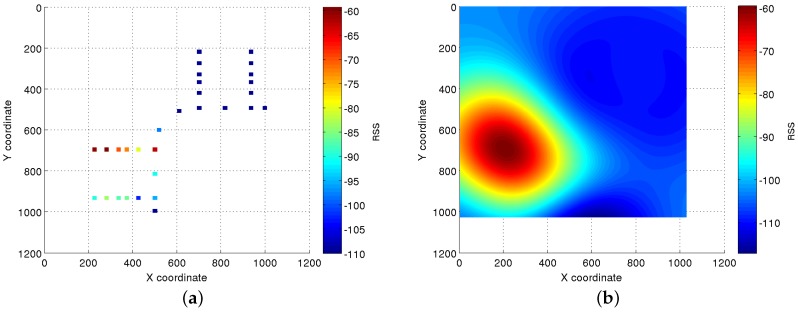
Discrete information and continuous reference surface estimated for an AP. (**a**) Discrete information matrix; (**b**) estimated RSS reference surface.

The goal is to obtain a reference surface for each AP containing the RSS expected at each coordinate of the environment by using an SVR algorithm. For each AP, the available training data will be assigned to the corresponding coordinate, and then, the missing RSSs will be inferred using SVR. The training stage consists of the following steps:

- Creation of an RSS map for each AP using discrete information:
  First, the environment is site-surveyed as in the usual fingerprint-based method: RSS is measured at some positions of the environment and stored in the fingerprint database FP (Equation (1)).
  (1) $$\mathrm{FP}=\left(\begin{array}{cccc} RSS_{AP_{1}}\left(P_{1}\right) & RSS_{AP_{2}}\left(P_{1}\right) & \ldots & RSS_{AP_{n}}\left(P_{1}\right)\\ RSS_{AP_{1}}\left(P_{2}\right) & RSS_{AP_{2}}\left(P_{2}\right) & \ldots & RSS_{AP_{n}}\left(P_{2}\right)\\ \vdots & \vdots & \ddots & \vdots \\ RSS_{AP_{1}}\left(P_{p}\right) & RSS_{AP_{2}}\left(P_{p}\right) & \ldots & RSS_{AP_{n}}\left(P_{p}\right) \end{array}\right)$$
  where $RSS_{AP_{1}}\left(P_{1}\right)$ is the RSS from $AP_{1}$ at Position 1, *n* is the number of APs and *p* is the number of site-surveyed positions.
  Then, *n* matrices $RSS_{AP_{i}}$ (one per AP) are created using the information contained in the fingerprint database (Equation (2)).
  (2) $$RSS_{AP_{i}}=\begin{array}{cc} & \begin{array}{cccc} y_{1} & y_{2} & \ldots & y_{p_{2}} \end{array}\\ \begin{array}{c} x_{1}\\ x_{2}\\ \vdots \\ x_{p_{1}} \end{array} & \left(\begin{array}{cccc} RSS(x_{1},y_{1}) & RSS(x_{1},y_{2}) & \ldots & RSS(x_{1},y_{p_{2}})\\ RSS(x_{2},y_{1}) & RSS(x_{2},y_{2}) & \ldots & RSS(x_{2},y_{p_{2}})\\ \vdots & \vdots & \ddots & \vdots \\ RSS(x_{p_{1}},y_{1}) & RSS(x_{p_{1}},y_{2}) & \ldots & RSS(x_{p_{1}},y_{p_{2}} \end{array}\right) \end{array}$$
  where $RSS(x_{1},y_{1})$ is the RSS from $AP_{i}$ at coordinate $\left(x_{1},y_{1}\right)$ if the coordinate $\left(x_{1},y_{1}\right)$ was site-surveyed and unknown otherwise. In the case the coordinate $\left(x_{1},y_{1}\right)$ was site-surveyed, but no signal was received from $AP_{i}$, the $RSS(x_{1},y_{1})$ value is set to a minimum value (−110 dBm in our experimentation). This way, the important information obtained when an AP is not detected at a certain coordinate can be used to correctly estimate the RSS at non-site-surveyed coordinates. $p_{1}$ is the number of coordinates represented in the *x* axis, and $p_{2}$ is the number of coordinates represented in the *y* axis. The number of coordinates $p_{1}$ and $p_{2}$ will be selected depending on the chosen resolution for the localization system. An analysis of the effect of changing the system resolution will be exposed in Section 4.3.3.
  An example of this matrix is represented in Figure 2a, where the colored dots represent the RSS at the site-surveyed coordinates, and the white background covers the coordinates with unknown RSS.
- Estimation of the continuous reference surfaces:
  The continuous reference surfaces are created using the information contained in the discrete matrices defined in the previous step. To do so, an *ϵ*-SVR algorithm [46] is used to infer the RSS values for the coordinates with unknown values.
  This process is performed in a two-step approach:
  First, *ϵ*-SVR is used to create a function $f_{AP_{i}}\left(v\right)$ (Equation (3)) for each $AP_{i},\;\mathrm{with},\;i=1,\cdots ,n$, that is able to estimate the target outputs for the input training data v with at most *ϵ* deviation.
  For a given training dataset $v=v_{AP_{i}}=\left\{\left(v_{1},z_{1}\right),\;\cdots ,\;\left(v_{p},z_{p}\right)\right\}$ for each AP, the function for linear SVR is defined as:
  (3) $$f_{AP_{i}}\left(v\right)=\langle \omega ,v\rangle +b$$
  where *b* is the “bias” term, *ω* is the normal vector to the SVR hyperplane (optimized by Equation (5)), $v_{j}$ is a feature vector (the coordinate (x,y) of position $P_{j}$) and $z_{j}$ is the target output (the RSS from $AP_{i}$ at position $P_{j}$) with,j=1,⋯,p.
  Then, the RSS expected to be measured in the coordinates with unknown values are estimated using this function. The resulting continuous surfaces can be seen as maps of estimated coverage for each AP.
  Under the selection of the parameters ϵ>0 and C>0 (*C* is the regularization parameter, determining the trade-off between training error and model complexity), the function is obtained by:
  (4) $$\begin{array}{cc} \underset{\omega ,\;b,\;\xi_{j},\;\xi_{j}^{*}}{\mathrm{minimize}} & \frac{1}{2}{∥\omega ∥}^{2}+C\sum_{j=1}^{p}\left(\xi_{j}+\xi_{j}^{*}\right)\\ \mathrm{subject}\;\mathrm{to} & z_{j}-\langle \omega ,v\rangle -b\;\leq \;\epsilon +\xi_{j}\\ & \langle \omega ,v\rangle +b-z_{j}\;\leq \;\epsilon +\xi_{j}^{*}\\ & \xi_{j},\;\xi_{j}^{*}\;\geq \;0 \end{array}$$
  dealing with the so-called *ϵ*-insensitive loss function ${\left|\xi \right|}_{\epsilon }$ described by:
  (5) $${\left|\xi \right|}_{\epsilon }:=\left\{\begin{array}{ccc} 0 & & if\;\left|z-f(v)\right|\leq \epsilon \\ \left|\;z-f(v)\right|-\epsilon & & otherwise. \end{array}\right.$$
  This optimization problem is computationally simpler to solve when transformed into its Lagrange dual formulation (called the dual problem). The dual formula is obtained introducing the $\alpha_{j}$ and $\alpha_{j}^{*}$ multipliers by:
  (6) $$\begin{array}{cccc} & \mathrm{minimize} & & L\left(\alpha \right)=\frac{1}{2}\sum_{j=1}^{p}\sum_{k=1}^{p}\left(\alpha_{j}-\alpha_{j}^{*}\right)\left(\alpha_{k}-\alpha_{k}^{*}\right)\langle v_{j},v_{k}\rangle +\epsilon \sum_{j=1}^{p}\left(\alpha_{j}+\alpha_{j}^{*}\right)-\sum_{j=1}^{p}z_{j}\left(\alpha_{j}^{*}-\alpha_{j}\right)\\ & \mathrm{subject}\;\mathrm{to} & & \sum_{j=1}^{p}\left(\alpha_{j}-\alpha_{j}^{*}\right)=0\\ & & & 0\leq \alpha_{j}\leq C\\ & & & 0\leq \alpha_{j}^{*}\leq C \end{array}$$
  The ***ω*** parameter can be described as a linear combination of the training observations using the equation:
  (7) $$\omega =\sum_{j=1}^{p}\left(\alpha_{j}-\alpha_{j}^{*}\right)z_{j}$$
  The function $f_{AP_{i}}\left(v\right)$ for the dual problem is therefore:
  (8) $$f_{AP_{i}}\left(v\right)=\sum_{j=1}^{p}\left(\alpha_{j}-\alpha_{j}^{*}\right)\langle z_{j},z\rangle +b$$
  The bias term can be computed by using the Karush–Kuhn–Tucker (KKT) [47,48] conditions. These conditions state that the product between dual variables and constraints has to disappear at the optimal solution, obtaining the following conditions:
  (9) $$\begin{array}{c} \alpha_{j}\left(\epsilon +\xi_{j}-z_{j}+\langle \omega ,v_{j}\rangle +b\right)=0\\ \alpha_{j}^{*}\left(\epsilon +\xi_{j}^{*}+z_{j}-\langle \omega ,v_{j}\rangle -b\right)=0\\ \left(C-\alpha_{j}\right)\xi_{j}=0\\ \left(C-\alpha_{j}^{*}\right)\xi_{j}^{*}=0 \end{array}$$
  According to these conditions, *b* can be computed as follows:
  (10) $$\begin{array}{ccc} b=z_{j}-\langle \omega ,v_{j}\rangle -\epsilon & & \mathrm{for}\;\alpha_{i}\in \left(0,C\right)\\ b=z_{j}-\langle \omega ,v_{j}\rangle +\epsilon & & \mathrm{for}\;\alpha_{i}^{*}\in \left(0,C\right) \end{array}$$
  Finally, the dual formula is extended to support nonlinear functions by replacing the dot product $\langle v,v^{\prime }\rangle$ with a nonlinear kernel function $k\left(v,v^{\prime }\right)=\langle \Phi \left(v\right),\Phi \left(v^{\prime }\right)\rangle$, where Φ(v) is used to transform v to a high-dimensional space. This way, the original problem comes to:
  (11) $$\begin{array}{cccc} & \mathrm{minimize} & & L\left(\alpha \right)=\frac{1}{2}\sum_{j=1}^{p}\sum_{k=1}^{p}\left(\alpha_{j}-\alpha_{j}^{*}\right)\left(\alpha_{k}-\alpha_{k}^{*}\right)k\left(v,v^{\prime }\right)+\epsilon \sum_{j=1}^{p}\left(\alpha_{j}+\alpha_{j}^{*}\right)-\sum_{j=1}^{p}z_{j}\left(\alpha_{j}^{*}-\alpha_{j}\right)\\ & \mathrm{subject}\;\mathrm{to} & & \sum_{j=1}^{p}\left(\alpha_{j}-\alpha_{j}^{*}\right)=0\\ & & & 0\leq \alpha_{j}\leq C\\ & & & 0\leq \alpha_{j}^{*}\leq C \end{array}$$
  and the function for the nonlinear SVR used to estimate the output values is as follows:
  (12) $$f_{AP_{i}}\left(v\right)=\sum_{j=1}^{p}\left(\alpha_{j}-\alpha_{j}^{*}\right)k\left(v_{j},v\right)+b$$
  In this work, we have used the Radial Basis Function kernel (RBF kernel), which is defined as:
  (13) $$k\left(v,v^{\prime }\right)=exp\left(-\frac{{∥v-v^{\prime }∥}^{2}}{2\sigma^{2}}\right)$$
  where ${∥v-v^{\prime }∥}^{2}$ is the squared Euclidean distance between the two feature vectors and *σ* is a configuration parameter.
  Figure 2b shows the reference surface created using the *ϵ*-SVR algorithm trained with the AP discrete information depicted in Figure 2a. In this new surface, each coordinate contains the RSS expected to be measured from that coordinate.

### 3.2. Localization Stage

**Figure 3:**
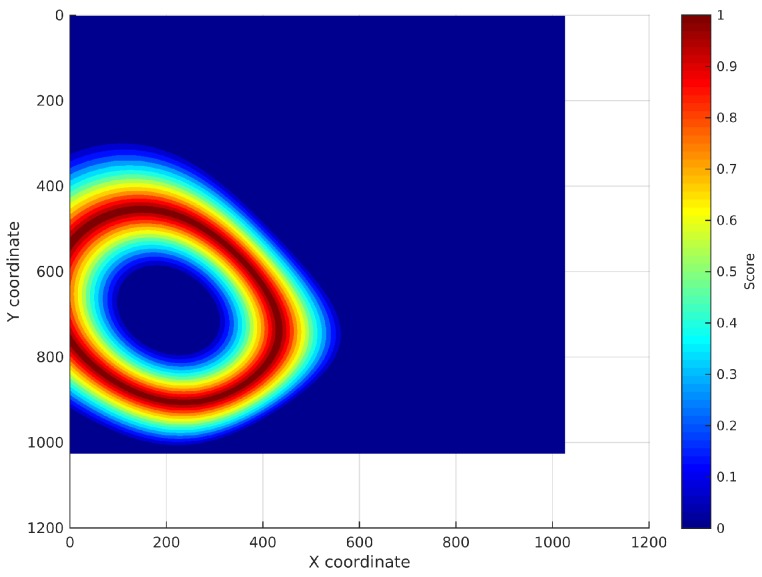
Localization subsurface for an AP: possible coordinates having a certain RSS collected at an unknown coordinate.

**Figure 4:**
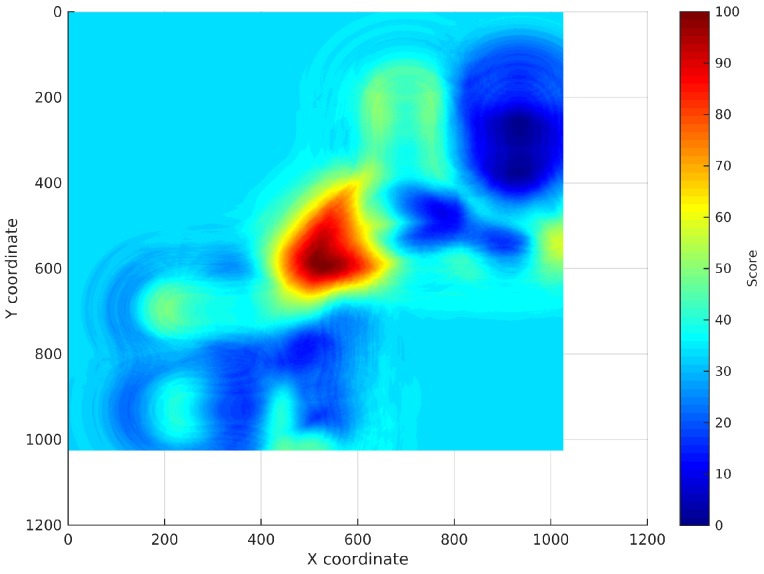
Resulting surface: sum of all of the APs’ localization subsurfaces for a test sample collected at an unknown coordinate.

**Figure 5:**
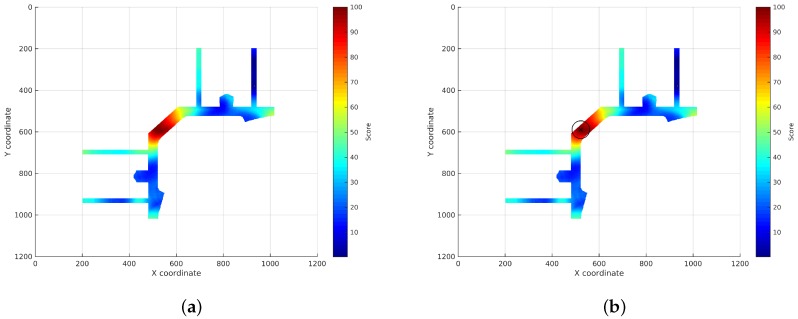
Resulting surface applying a mask of the environment to allow only reachable coordinates. (**a**) Masked surface to the reduce allowed locations; (**b**) location of the device.

In this stage, the objective is to identify the coordinate where the device has the highest probability to be. The location of the device will be obtained looking for the RSS from all APs in the continuous reference surfaces created during the training stage. The localization stage comprises six steps, as shown in Figure 1:

- Measurement of the RSS:
  An RSS sample is collected from every AP using the WiFi device to be located.
- Search for the RSSs in the continuous surfaces:
  The RSS from each AP is searched in the reference surface corresponding to that AP. This search is performed adding a noise margin to the RSS to deal with the noise characteristic of WiFi technology. An analysis of how this margin has been selected will be exposed in Section 4.3.1. The coordinates where the RSS is exactly the same as the RSS sample will obtain the maximum score, then the score will decrease as the RSS differs from the RSS sample until the difference between both values is higher than the margin. The rest of coordinates will get no score. This way, a new localization subsurface for each AP is created containing the scores for the coordinates that can be the location of the device (represented from light blue to red depending on their score in Figure 3). Figure 3 shows the subsurface obtained for the estimated reference surface shown in Figure 2b and an RSS of −97 dBm.
- Addition of the localization subsurfaces:
  The localization subsurfaces generated for each AP are now summed to obtain a new surface with higher values in the coordinates with higher probabilities of being the location of the device. An example of the results surface can be seen in Figure 4. In this surface, the red coordinates are more likely to be the location of the device.
- Application of an environment mask:
  The surface generated in the previous step can contain inaccessible areas outside the building. In addition, in certain applications, only certain areas of the environment are reachable for users. For instance, a museum visitor cannot access the storage rooms. To remove these unreachable areas, a mask is applied to the surface obtaining a new surface where only the reachable coordinates have a score (Figure 5a).
- Estimation of the device location:
  Finally, the location of the device will be estimated as the coordinate with the highest score in the resulting masked surface (Figure 5b).

## 4. Experimental Analysis

This section describes the experimental results reported using the continuous space estimator described in the previous section. A critical discussion of the results is provided.

### 4.1. Experimental Setup

**Figure 6:**
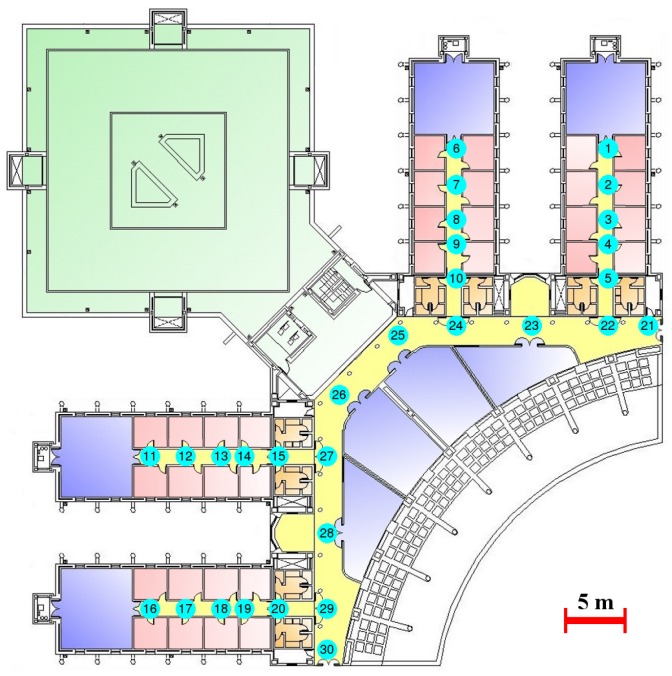
University of Alcalá (UAH) test-bed environment (30 topological positions).

The proposed WiFi localization system has been tested in a complex real-world environment with a surface of 3000 m${}^{2}$. The experiments have been performed on the third floor of west wing of the Polytechnic School at the University of Alcalá (UAH) (Figure 6). In this building, mainly made of concrete, the signal measurement is highly affected by the multipath effect [49] (reflection, refraction, shadowing and scattering, which causes a deviation from the original transmission to the device). In the experiments, 105 APs, deployed over the environment with the aim of providing Internet access to the students, but disregarding localization purposes, have been detected. Measurements collected at 30 significant topological positions have been considered to train the system (circled numbers in Figure 6). At each position, 30 fingerprints have been collected and averaged. As a consequence, one fingerprint per topological position has been used as training data. The continuous reference surfaces have been created using the averaged RSS at the 30 topological positions for all of the existing APs.

The tests have been carried out using two different approaches: first, using data collected standing at each topological position. In this approach, 30 fingerprints have been collected at each topological position and each of them have been used without averaging as test data. Second, data collected while a person holding a laptop was following different trajectories over the environment are used. Both kinds of data were collected on a different day and time than the training data, using the internal wireless interface of a laptop acquiring at its maximum allowed rate (one sample per second).

**Figure 7:**
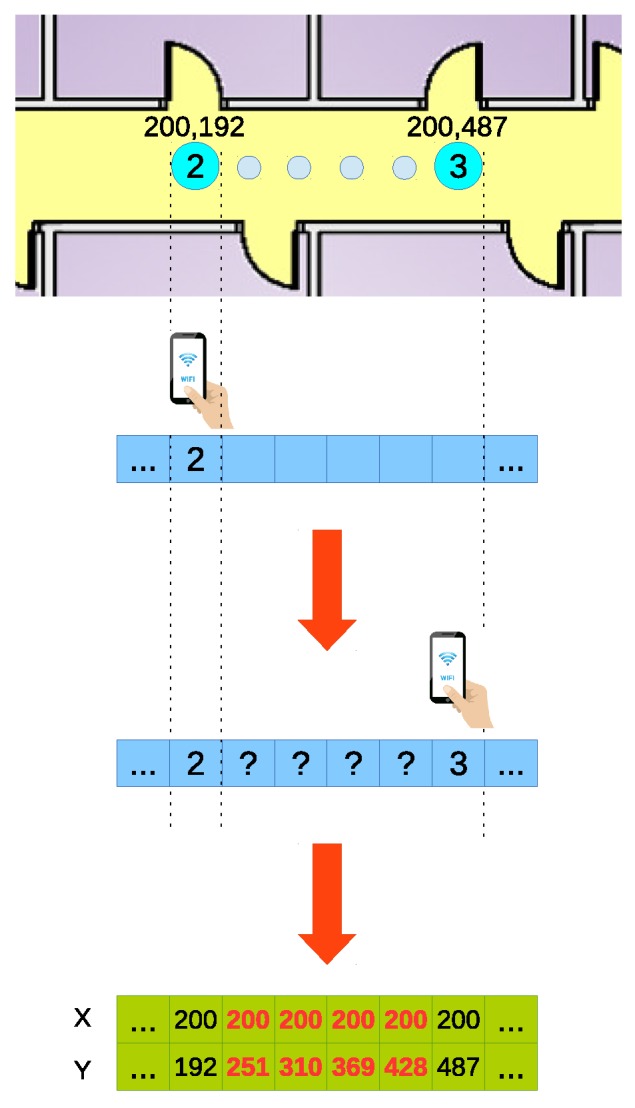
Ground truth generation.

The ground truth for the trajectories has been manually tagged: when the person carrying the device steps over a position existing on the training dataset, the sample is marked with the corresponding number of the position. With the assumption that a person walks at a constant speed of 1 m/s, each trajectory will be composed of samples measured one meter apart from each other. This means that most of the samples are measured in positions not covered in the training data and, as a consequence, with unknown coordinates. To tag these samples, we assumed that the user performing the test moves at a constant speed when moving from one known position to the next one. This way, the coordinates of the untagged samples are calculated by dividing uniformly the trajectory between two positions in a number of sections equal to the number of untagged samples (Figure 7).

This ground truth is used to calculate the mean distance error of the trajectory tests. The distance error is computed as the distance between the estimated coordinate and the target coordinate in the ground truth.

Finally, the results obtained using the proposed method will be compared with three of the most common methods used for indoor WiFi localization: RADAR [14], which uses K-Nearest Neighbors (KNN) [50] and is commonly used as the baseline to compare with new indoor WiFi localization methods [34,51]; Support Vector Machine (SVM) [52], which is based on the same principles as SVR, but for classification purposes; and finally, the method proposed by [53], which uses Random Forest (RF) [54] and is recognized by its high accuracy. These methods were implemented using the KNN, SVM and RF algorithm versions provided by the data mining tool Weka [55,56].

### 4.2. Experimental Results

This section describes the results of the experiments locating a WiFi device at the discrete positions used to create the reference surfaces and in motion.

#### 4.2.1. Topological Positions

The results shown in this section were obtained using the test data collected at the topological positions defined in Section 4.1.

Table 1 summarizes the results obtained with the proposed method (CSE) compared with the results obtained using the RF [53], SVM and RADAR [14] algorithms.

The localization error is not improved using the CSE method in comparison with the RF, SVM and RADAR methods when locating the device at the positions covered in the radio map. This makes sense, since RF, SVM and RADAR are trained to only distinguish between the topological positions, while CSE is trained to be able to locate the device at all of the accessible coordinates. Even in this case, the mean error of the proposed method is equal to the mean error of SVM and better than the RADAR algorithm, the CSE mean error being 0.70 cm worse than the mean error using the RF-based algorithm. However, if the CSE resulting coordinate is replaced by the coordinate of the closest topological position, the mean error using the CSE algorithm is reduced to 0.98 m. Using this approximation, the mean error can be compared when all of the algorithms are able to only distinguish between the topological positions (topological CSE is, in this case, 36% better than the SVM algorithm and 18% worse than RF).

**Figure 8:**
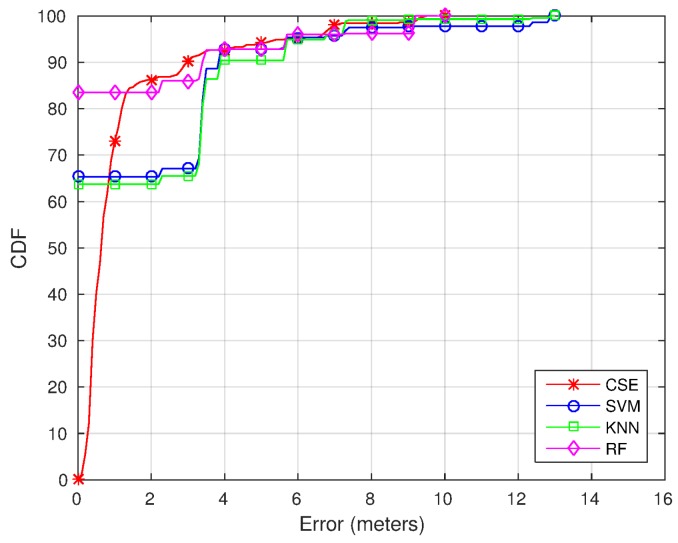
CDF using the topological positions dataset.

To fully understand the results, Figure 8 shows a comparison of the Cumulative Distribution Function (CDF) using CSE, RF, SVM and RADAR. The CDF shows an analysis of the distance to the ground truth coordinate for the different classifiers. As can be seen, the error is lower using CSE (except for the samples when RF, SVM or RADAR locate the device as the correct position, where the error is zero). The error is under 2 m for the 86th percentile using the CSE algorithm, under 2 m for the 83th percentile using the RF-based algorithm and under 2 m for the 65th percentile for the SVM and RADAR methods.

#### 4.2.2. Trajectories

**Figure 9:**
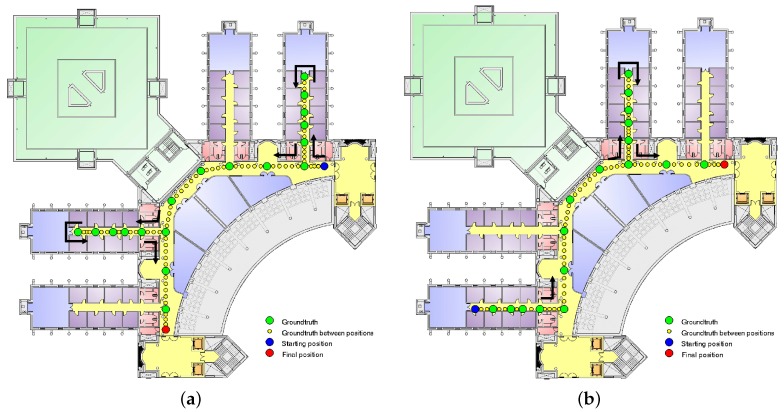
Trajectories used to test the proposed CSE method. (**a**) Trajectory 1; (**b**) Trajectory 2.

The results shown in this section were obtained during two different illustrative trajectories (Figure 9) covering all of the positions in the UAH environment to test the behavior of the system using the continuous space estimator.

On the first trajectory, a user was walking along the main, second and fourth corridors in a path of approximately 120 m at a mean speed of 0.73 m/s. Figure 9a shows the trajectory followed during the first experiment. This trajectory starts at the position marked with a blue circle and continues along the green and yellow dot path to the final position marked with a red circle. Green circles indicate the site-surveyed positions, while the yellow dots are locations where there are available measurements not corresponding to a position in the fingerprint database. On the second trajectory, the user was walking along the main, first and third corridors in a path of approximately 100 m at a mean speed of 0.73 m/s. This trajectory followed the opposite direction to the previous one along the main corridor; this way, all of the positions are covered at least twice with different orientations. Figure 9b shows the second trajectory with the same format as in Figure 9a.

Table 2 summarizes the results obtained with the different classifiers using the trajectories dataset. As can be seen, the mean distance error is highly reduced using the proposed CSE.

The best results when locating a device in motion are obtained using the proposed method. In this case, the mean error is reduced around 62% for the first trajectory and around 67% for the second trajectory compared to the second best algorithm (RF and SVM respectively). This way, the objective of the CSE is accomplished: the resolution is increased without increasing the effort building the fingerprint database.

**Figure 10:**
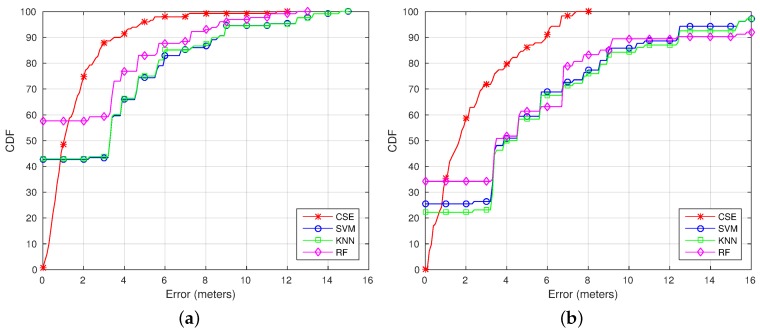
CDF using the trajectories dataset. (**a**) Trajectory 1; (**b**) Trajectory 2.

Figure 10 shows the CDF for Trajectories 1 (Figure 10a) and 2 (Figure 10b). As can be seen looking at the CDFs, the error is lower using CSE most of the time (again, except for the samples when RF, SVM or RADAR locates the device as the correct position, where the error is zero). For Trajectory 1, the error is reduced from 3.85 to 2 m for the 75th percentile and from 7.25 to 3.5 m for the 90th percentile. The same way, for Trajectory 2, the error is reduced from 6.8 to 3.32 m for the 75th percentile and from 12.35 to 5.92 m for the 90th percentile.

### 4.3. Parameters and Resolution Evaluation

This section goes in depth with the analysis performed to evaluate the validity of the proposed method, including the effect of changing the noise margin to create the localization subsurfaces, the resolution of the positions used as training data and the resolution of the reference surfaces.

#### 4.3.1. Noise Margin Evaluation

This section describes the effect of changing the noise margin applied while creating the localization subsurfaces as explained in Section 3.2. The noise margin has been initially set at 10 dB, since it is the signal variation caused by the so-called temporal variations [57], which are the RSS variations due to WiFi noise and non-controllable changes in the physical environment. However, the effect of changing this parameter is analyzed in order to check if this value is the correct choice.

**Figure 11:**
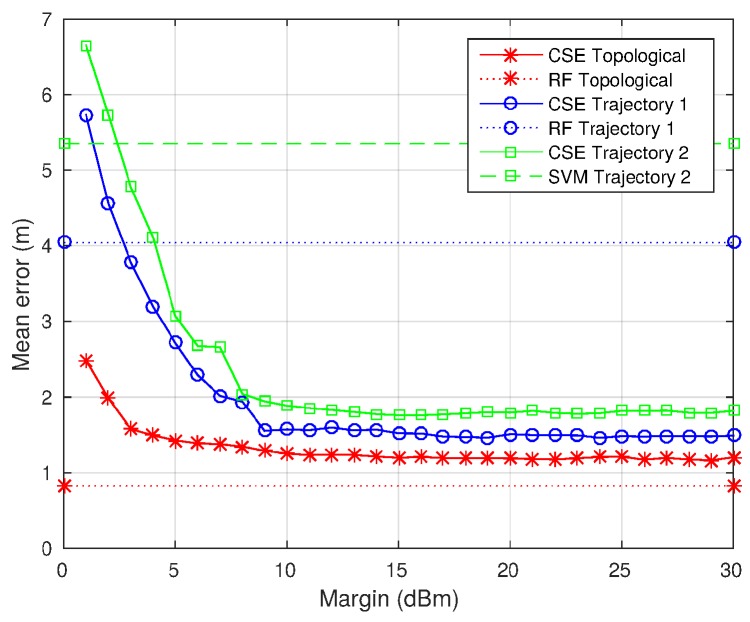
Mean error evolution changing the noise margin used to create the localization subsurfaces.

Figure 11 shows the mean error obtained using margin errors from 1 dB to 30 dB compared with the algorithm providing the lowest mean error (among RF, SVM and RADAR). As can be seen, mean error decreases as the noise margin is increased until a margin around 10 dB is reached, then from 11 to 30 dB, the mean error remains almost constant. Looking at the trajectories results, it can be seen that for any margin above 5 dB, the CSE algorithm provides lower errors than the rest of the algorithms. From this analysis, it can be stated that a margin error of 10 dB or above is sufficient to provide the best results.

**Figure 12:**
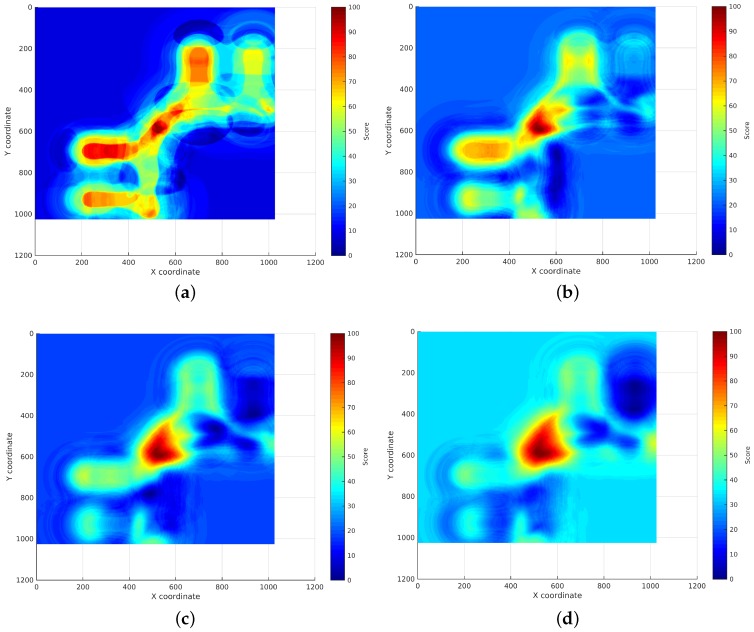
Localization subsurfaces created using different noise margins. (**a**) Margin 1; (**b**) Margin 5; (**c**) Margin 10; (**d**) Margin 15.

Finally, Figure 12 shows examples of the localization subsurfaces obtained using different margin errors, namely 1, 5, 10 and 15 dB. As can be seen, as the margin is increased, the areas with higher scores (orange-red ones) are reduced, obtaining one high-scored area for margins above 10 dB and, as a consequence, reducing the uncertainty for the device position.

#### 4.3.2. Training Resolution

This section describes the results of the experiments performed changing the number and location of the topological positions used as training data. The objective is to assess the effectiveness of the proposed system when the number of positions with available information to train the system is reduced. This analysis is performed using two different approaches: On the one hand, the system is trained using manually selected configurations composed of uniformly-distributed positions. On the other hand, the system is trained using randomly-selected configurations.

**Figure 13:**
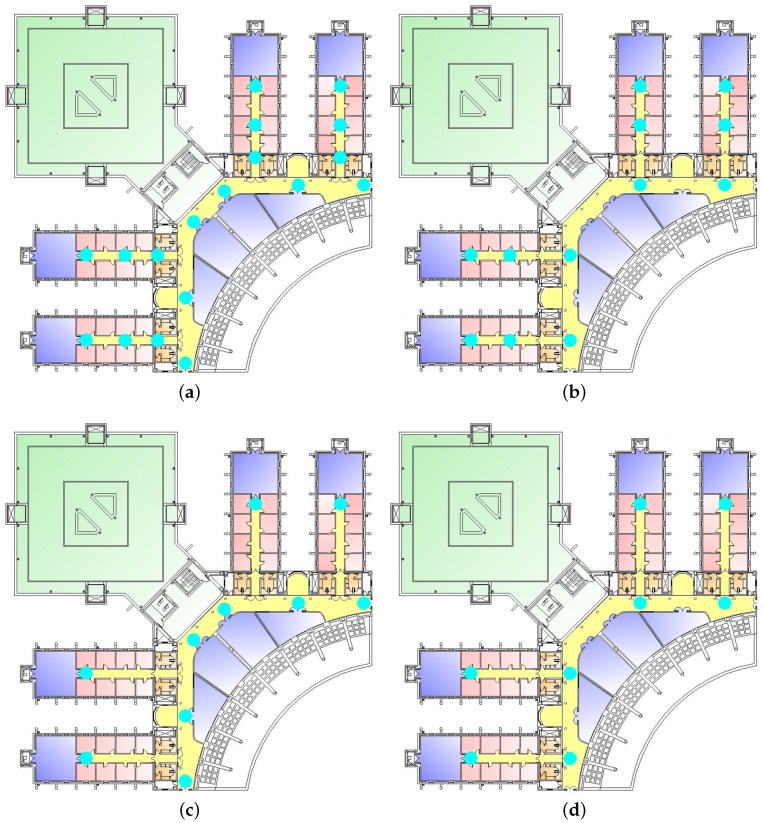
Manually selected configurations for training resolution experimentation. (**a**) Configuration 1: 18 positions; (**b**) Configuration 2: 12 positions; (**c**) Configuration 3: 10 positions; (**d**) Configuration 4: eight positions.

Figure 13 shows the different manually-selected configurations used to train the system. These configurations have been chosen by selecting the topological positions located at the beginning, center and end of the corridors for Configurations 1 and 2 (Figure 13a,b). Then, Configuration 3 (Figure 13c) has been created removing some positions from Configuration 1 and Configuration 4 (Figure 13d) removing some positions from Configuration 2.

Table 3 shows the number of positions, minimum distance and mean distance between two positions for the different configurations. The datasets used to test the different configurations are the ones used in the previous experiments (as explained in Section 4.1).

Table 4 summarizes the results obtained when training the system using the data available in the different configurations for the trajectories’ test dataset. As can be seen, CSE provides the best results for all of the configurations used to train the system. Even more, no matter the configuration selected to train the CSE, it provides lower mean errors than the best configuration for the rest of the algorithms: For Trajectory 1, in the worst case, if the system is trained using the eight-position configuration, CSE provides a mean error of 4.02 m, which is still better than the lowest error using one of the other algorithms (RF) with the 30-position configuration (getting a mean error of 4.04 m). The same way, for Trajectory 2, CSE gets a mean error of 3.57 m using the eight-position configuration, while the mean error is 5.35 using the 30-position training dataset and the SVM classifier.

In general, the lower the number of positions in the training dataset the higher the mean error of the system. This is the expected behavior; however, when using Configuration 1, the mean error is not significantly increased compared to the mean error using the original configuration, but the site survey effort is reduced to almost half. Using this configuration, the number of positions to the site survey is reduced from 30 positions to 18 without significantly increasing the mean error.

**Figure 14:**
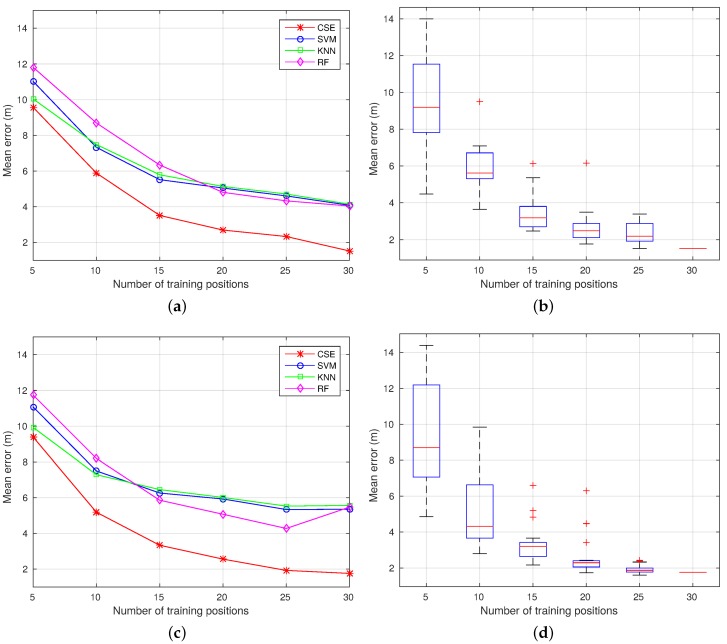
Mean error evolution changing the number of randomly-selected training positions. (**a**) Trajectory 1: mean error; (**b**) Trajectory 1: CSE boxplot; (**c**) Trajectory 2: mean error; (**d**) Trajectory 2: CSE boxplot.

To complement these results, 20 random configurations have been selected to train the system using 5, 10, 15, 20 and 25 positions. This way, it can be proven that the proposed algorithm increases the localization accuracy, while reducing the site survey effort independently of how well the training positions have been chosen. A comparison of the mean error obtained when considering all of the algorithms is shown in Figure 14a,c for Trajectories 1 and 2 respectively. In addition, the dispersion for each group of configurations is shown using boxplots in Figure 14b,d for Trajectories 1 and 2, respectively. Looking at these figures, it can be seen that CSE provides the lowest error independently of the number of positions available in the training dataset. Moreover, in both experiments, the results obtained using CSE trained with configurations containing 15 or more positions are better than any configuration using RF, SVM or RADAR, including those with a higher number of positions in the training dataset.

Regarding the CSE results, the higher the number of positions in the training dataset, the more compact the mean errors, as can be seen in the boxplot figures. This means that the selection of the training positions is more critical when the number of positions in the training dataset is smaller. Finally, after analyzing the configurations providing the best and worst results, it can be concluded that the more uniformly distributed the selected positions, the lower the mean error of the proposed system.

#### 4.3.3. Reference Surfaces Resolution

This section describes the results obtained when changing the number of coordinates to cover the environment. Each coordinate will cover a squared area of the environment. As a consequence, the higher the number of coordinates, the smaller the area covered by each one of them.

**Figure 15:**
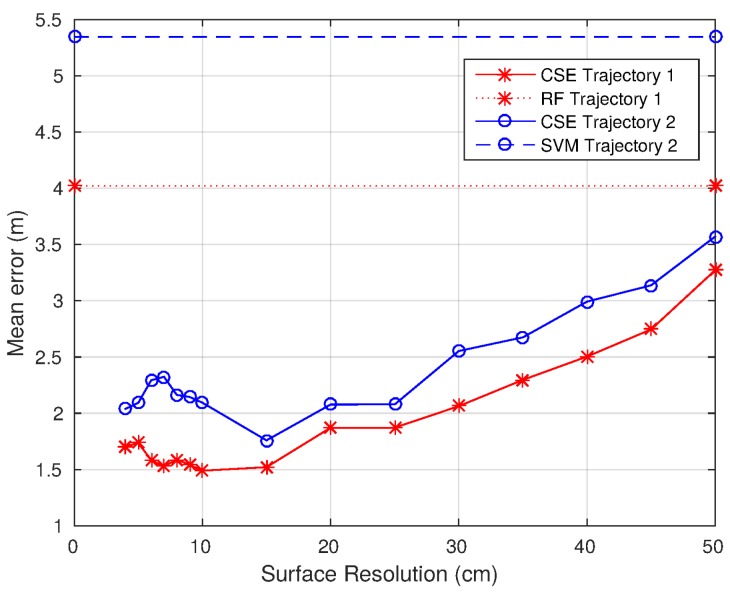
Mean error evolution changing the reference surfaces’ resolution.

Figure 15 shows the mean error evolution with the size of the coordinates. As can be seen, the mean error decreases with the area covered by each coordinate until the coordinates are as small as 15 cm on the side. From the coordinates of the 15-cm side to the 4-cm side, the error increases. As a consequence, 15 cm-sided coordinates have been chosen for the experimentation. These results are consistent with the WiFi signal behavior, widely covered in the literature. The small-scale variations cause the RSS to vary when the WiFi device moves distances in the range of the wavelength [57]. This effect makes it very difficult to estimate the correct location because small variations in the position can lead to high RSS variations. For the 802.11 b/g networks, working at 2.4 GHz, the wavelength is 12.5 cm. This fact supports the conclusion drawn from the results: the lowest main error is achieved with the highest resolution provided that the coordinates’ size is bigger than the wavelength.

Finally, even though the selection of the reference surfaces resolution can be a determinant for the localization accuracy of the system (being able to reduce the mean error by more than 50%), this selection is not critical when compared with other localization algorithms, as the CSE is able to increase accuracy independently of the resolution used to represent the continuous localization reference surfaces.

### 4.4. Statistical Validation of the Reported Results

With the aim of validating statistically the results reported in previous sections, we have set the next null hypothesis ($H_{0}$):

- Ranking: The means of the results achieved by two or more methods under consideration (CSE, RF, SVM and RADAR) are the same.
- Post-hoc with control method: The mean of the results of the control method and against each other group is equal (compared in pairs).

Given the nature of our experiments (number of groups *k* = 4, number of samples *n* = 11, paired data, normality and homoscedasticity not satisfied), we have chosen first Friedman aligned ranks [58,59] as the ranking test and then Holm [60] as the post-hoc test. In addition, our proposal, CSE, is considered as the control method with a significance level (*α*) equal to 0.05.

The null hypothesis for ranking was rejected by the Friedman aligned ranks test with a *p*-value equal to 0.00012. Moreover, the null hypothesis for the post-hoc test was also rejected for the three pairs’ comparisons:

- CSE vs. RF: $H_{0}$ is rejected with the *p*-value equal to 0.00001.
- CSE vs. SVM: $H_{0}$ is rejected with the *p*-value equal to 0.00114.
- CSE vs. RADAR: $H_{0}$ is rejected with the *p*-value equal to 0.00061.

Notice that the tests cited above were applied with the STAC web platform [61], and they validated the fact that the results reported by CSE were statistically significant against those reported by the methods considered for comparison purposes.

## 5. Conclusions and Future Work

This work has presented a method to increase fingerprint-based WiFi localization resolution without increasing the site survey effort. To do so, a continuous space estimator to cover positions not stored in the fingerprint database has been developed. This continuous space estimator uses an SVR algorithm to estimate virtual fingerprints not available in the fingerprint database. First, a continuous reference surface for each AP in the environment is created using the discrete fingerprints and SVR to estimate the RSS at non-site-surveyed positions. Then, the RSSs received during the localization stage are searched in these surfaces. This way, the position of the device can be more accurately estimated, being able to provide localization using a continuous map representation without increasing the cost of building the fingerprint database. It is important to highlight that it is not necessary to know where the APs are located to deploy the localization system. This aspect is especially interesting regarding its deployment in new unknown environments.

The proposal was tested in a real-world environment considering two different approaches. The first one was localization at the static positions of the environment covered in the fingerprint database, while the second one was localization of a device in motion. The aim of using two different approaches was to show how the proposed method was able to reduce the mean error when locating a device at unknown positions, while not increasing the error at the known ones.

In light of the results, we can conclude that our proposal successfully increases resolution without increasing the site survey effort. The mean distance error was reduced more than 60% in comparison to a state-of-the-art traditional fingerprint localization method when locating a device in motion. At the same time, the mean error was not significantly increased when locating the device at static known positions.

Moreover, the analysis of the number and distribution of the positions needed to train the system showed that the number of site-surveyed positions can be reduced to almost half without significantly increasing the mean distance error if the positions chosen to train the system are uniformly distributed over the environment. However, a very interesting research line for the future could include a method to select the optimal distribution and number of positions to site survey. This method could help to reduce even more the site survey effort obtaining minimal mean localization errors.

In the future, the proposed continuous space estimator will be also improved by using the hierarchical localization approach developed by the authors in [62]. In addition, a procedure for selecting some of the APs will be tested. This way, the effort needed to estimate the continuous reference surfaces and to search for the RSS during the localization stage could be reduced.

We are also planning on including the information provided by other sensors, such as Li-Fi, accelerometers or compass, to improve the proposed localization system during localization of a device in motion. The use of Li-Fi technology is likely to improve the performance of our system in the near future, but further experimental analysis is required. In addition, by knowing the direction of movement or even if the device is moving or standing at a position, the trajectory of the device could be filtered to avoid location estimations too far away from the previous ones.

Finally, the proposed method has been proven useful to reduce the site survey effort, but has not eliminated it. As a consequence, an interesting research area could be focused on designing an automated fingerprint database generation process and a maintenance system to keep the database up to date using the information collected during the localization stage.

## Figures and Tables

**Table 1:** Experimental results: test using the topological positions dataset. CSE, Continuous Space Estimator.

	Mean Error			
	CSE	RF [53]	SVM	RADAR [14]
Topological positions	1.53 m	0.83 m	1.53 m	1.56 m

**Table 2:** Experimental results: test using the trajectory datasets.

	Mean Error			
	CSE	RF [53]	SVM	RADAR [14]
Trajectory 1	1.52 m	4.04 m	4.08 m	4.14 m
Trajectory 2	1.76 m	5.47 m	5.35 m	5.57 m

**Table 3:** Training resolution configurations.

	All Positions	Config. 1	Config. 2	Config. 3	Config. 4
Number of positions	30	18	12	10	8
Minimum distance between positions	2.23 m	5.50 m	6.80 m	8.00 m	14.53 m
Mean distance between positions	26.58 m	27.46 m	29.63 m	29.75 m	31.35 m

**Table 4:** Training resolution experimentation.

		Mean Error				
		All Pos.	Config. 1	Config. 2	Config. 3	Config. 4
Trajectory 1	CSE	1.52 m	1.71 m	3.38 m	3.39 m	4.02 m
	RF [53]	4.04 m	4.31 m	8.79 m	6.56 m	6.10 m
	SVM	4.08 m	4.93 m	5.16 m	5.97 m	5.63 m
	RADAR [14]	4.14 m	4.20 m	5.69 m	6.06 m	6.16 m
Trajectory 2	CSE	1.76 m	2.01 m	2.80 m	3.67 m	3.57 m
	RF [53]	5.47 m	5.67 m	9.18 m	8.43 m	5.87 m
	SVM	5.35 m	5.65 m	6.24 m	6.06 m	6.06 m
	RADAR [14]	5.57 m	5.90 m	6.40 m	6.45 m	5.94 m
